# Effect of Rubber Nanoparticle Agglomeration on Properties of Thermoplastic Vulcanizates during Dynamic Vulcanization

**DOI:** 10.3390/polym8040127

**Published:** 2016-04-15

**Authors:** Hanguang Wu, Ming Tian, Liqun Zhang, Hongchi Tian, Youping Wu, Nanying Ning, Guo-Hua Hu

**Affiliations:** 1 Key Laboratory of Beijing City on Preparation and Processing of Novel Polymer Materials, Beijing University of Chemical Technology, Beijing 100029, China; hanguangwu.1988@163.com (H.W.); zhanglq@mail.buct.edu.cn (L.Z.); 13964511068@163.com (H.T.); wuyp@mail.buct.edu.cn (Y.W.); 2 State Key Laboratory of Organic-Inorganic Composites, Beijing University of Chemical Technology, Beijing 100029, China; 3 Laboratory of Reactions and Process Engineering, University of Lorraine-CNRS, Nancy 54500, France; guo-hua.hu@univ-lorraine.fr

**Keywords:** thermoplastic vulcanizate, morphological evolution, tensile properties, elasticity, melt processability

## Abstract

We previously reported that the dispersed rubber microparticles in ethylene-propylene-diene monomer (EPDM)/polypropylene (PP) thermoplastic vulcanizates (TPVs) are actually agglomerates of rubber nanoparticles. In this study, based on this new understanding of the microstructure of TPV, we further revealed the microstructure-properties relationship of EPDM/PP TPV during dynamic vulcanization, especially the effect of the size of rubber nanoparticle agglomerates (*d*_n_), the thicknesses of PP ligaments (*ID*_poly_) and the rubber network on the properties of EPDM/PP TPV. We were able to simultaneously obtain a high tensile strength, elongation at break, elastic modulus, and elasticity for the EPDM/PP TPV by the achievement of a smaller *d*_n_, a thinner *ID*_poly_ and a denser rubber network. Interestingly, the effect of *d*_n_ and *ID*_poly_ on the elastic modulus of EPDM/PP TPV composed of rubber nanoparticle agglomerates is different from that of EPDM/PP TPVs composed of rubber microparticles reported previously. The deformation behavior of the TPVs during stretching was studied to understand the mechanism for the achievement of good mechanical properties. Interestingly, the rubber nanoparticle agglomerates are oriented along the tensile direction during stretching. The TPV samples with smaller and more numerous rubber nanoparticle agglomerates can slow down the development of voids and cracks more effectively, thus leading to increase in tensile strength and elongation at break of the EPDM/PP TPV.

## 1. Introduction

Thermoplastic vulcanizates (TPVs) are a group of high performance thermoplastic elastomers with a high content of crosslinked rubber as the dispersed phase and a low content of thermoplastics as the continuous phase [1]. TPVs combine the high elasticity of conventional vulcanized elastomers and the high processability and recyclability of thermoplastics. Thus, TPVs have attracted much attention as “green” polymers in recent years and have been widely used in industries such as automotive, construction, and electronics. Nowadays, TPVs are some of the fastest growing elastomers because of the requirement of environmental protection and resource savings [2].

TPVs are produced by dynamic vulcanization (DV), a complicated reactive blending process consisting of the breakup and crosslinking of the rubber phase and the phase inversion of the rubber and the thermoplastic, dominating the formation of phase structure including the rubber particle size, the rubber network, and the thicknesses of the matrix ligaments [1]. A high content (60–80 wt %) of rubber phase is generally required to obtain TPVs with high elasticity. The rubber particle size and the thicknesses of the matrix ligaments are believed to have significant effects on the properties of TPVs, such as the tensile properties, the elasticity and the melt processability [3,4,5,6,7,8]. For example, Coran *et al.* studied the effect of rubber particle size on the tensile properties of ethylene propylene diene monomer/polypropylene (EPDM/PP) blends produced by mixing pre-crosslinked rubber particles with different sizes into a PP matrix, and found that the tensile strength and elongation at break increased with the decrease in rubber particle size [7,8]. However, the vulcanized EPDM/PP blends they prepared have much lower tensile properties than those of the EPDM/PP TPVs produced by DV. L’Abee *et al.* prepared TPVs with different rubber particle sizes by varying the rotor speed of the batch mixer during DV. They studied the effect of the number-averaged size of the rubber particles on the tensile properties, elasticity and melt processability of the TPVs to provide guidance for controlling properties-processing balance of TPVs on their elasticity and the melt processability. The results showed that *d*_n_ is an important parameter to vary the mechanical properties and melt processability of TPV [8]. A decrease in *d*_n_ leads to an increase in tensile properties and elasticity, but a decrease in processability. The studies of Coran *et al.* and L’Abee *et al.* were focused on TPVs with rubber microparticles (*d*_n_ = 1–70 μm). However, the size of the rubber particles in commercial EPDM/PP TPVs ranges from 0.5 to 2.0 μm [2].

In our previous study, we observed the formation of rubber nanoparticles at the early stage of DV through the breakup of the rubber phase and the *in situ* vulcanization of the rubber droplets [9], and the subsequent agglomeration of these rubber nanoparticle into rubber micoparticles during DV [10]. Then, we revealed the relationship between the formation and agglomeration of the rubber nanoparticles and the variation of the rubber network during DV, which is expected to play an important role in the properties of TPVs. The effect of the rubber nanoparticle agglomerates (RNAs) on the properties of TPVs should be different from that of the rubber micoparticles reported in the previous studies. As far as we know, the variations of the properties of TPVs with the evolution of the morphology during DV have not been reported.

In this study, with the new understanding of the morphological evolution of TPV, we further studied the effect of RNAs on the properties of TPV during DV and the microstructure-properties relationship of EPDM/PP TPV during DV, especially the effect of the size of RNAs (*d*_n_), the thicknesses of PP ligaments (*ID*_poly_), and the density of the rubber network on the properties. This study aimed to provide guidance for the preparation of high performance TPV products by controlling the microstructure of TPVs during DV.

## 2. Experimental Section

### 2.1. Materials

The PP, (Melt flow index = 0.5 g·10 min^−1^, *T*_m_ = 159 °C), the EPDM rubber (ethylene content = 66%, Mooney Viscosity = 61 Pa·s (Test condition: Large rotor, 125 °C), the chemical structure has been reported previously[11]). The crosslinking system was composed of resol and SnCl_2_ supplied by XinJiang Karamay Oilfield (Xinjiang, China). A commercially available pentaerythritol tetrakys 3-(3,5-ditert-butyl-4-hydroxyphenyl) propionate (1010) was used as an antioxidant. [9].

### 2.2. Dynamic Vulcanization of EPDM/PP System

EPDM/PP TPV samples with 60 wt % EPDM were prepared in a Haake internal mixer (HAAKE Rheomix 600 OS, Thermo Fisher Scientific, Waltham, MA, USA). The premix compositing of the EPDM rubber phase, PP, antioxidant, and crosslinking system was fed into the Haake rheomix at 170 °C and a rotor speed of 80 rpm, which was described in our previous articles [9,10]. We chose EPDM/PP blend with single composition as an example to study the mechanism of the morphological evolution of TPV during DV because the change in the composition of TPV or the processing condition only affects the size of the rubber agglomerates and the rate of the morphological evolution, but not the relationship between morphology and properties of TPVs. The effect of the composition and processing condition on the morphological evolution of TPV will be studied in our future work.

In order to establish a relationship between the microstructures and properties of the TPV during DV, samples were taken at different mixing times in the late stage of DV and were immediately quenched in liquid nitrogen to freeze the morphology. Noted that the antioxidants were added in the EPDM/PP blends and the Haake internal mixer with the closed environment was used to ensure that the PP phase had no degradation during DV, as reported in previous studies [9,10].

### 2.3. Compression Molding of the Samples

The TPV samples taken from the Haake internal mixer were compression molded at 180 °C to prepare films of 1 mm thick for the following elasticity testing, tensile properties testing, and processability testing. In order to obtain the accurate morphology-properties relationship of TPVs during DV, the morphology of the compression-molded TPV samples were measured.

### 2.4. Characterizations

#### 2.4.1. Degree of Crosslinking of Rubber Phase

The volume swell ratio (*Q*) and the rubber gel content (% gel) of an EPDM/PP TPV were measured to quantify the degree of crosslinking of the EPDM phase. Rectangular pieces with dimensions of 10 × 10 × 2 mm^3^ were immersed in cyclohexane for 48 h under gentle stirring. The swollen samples were weighed, dried and weighed again. Drying of the samples was performed at 80 °C for 12 h to remove the solvent absorbed by the samples. The % gel and *Q* were calculated by the following equations [8,12]:

(1) $$Q=\frac{V}{V_{P}}=\frac{1}{\phi_{P}}$$

(2) $$\phi_{p}=\frac{V_{p}}{V}=\frac{1}{1+\frac{m_{s}-m_{d}}{m_{d}}\cdot \frac{\rho_{p}}{\rho_{s}}}$$

(3) $$\%gel=\frac{m_{d}-(m\times \phi_{\text{insol}})}{m\times \phi_{\text{rubber}}}\times 100\%$$

where *ρ*_p_ and *ρ*_s_ are the densities of the sample and the solvent (cyclohexane), respectively; *V*_p_ and *V* are the volumes of the sample before and after swelling, respectively; *m* is the mass of the sample before extraction; *m*_s_ and *m*_d_ are the masses of the swollen sample and dried sample after extraction, respectively; and $\phi_{\text{insol}}$ and $\phi_{\text{rubber}}$ are the mass fractions of the insoluble components (PP and crosslinked system) and EPDM, respectively.

#### 2.4.2. Morphology

The morphology of selected samples was examined by using peak force tapping atomic force microscopy (PF-AFM) (Nanoscope IIIa, Bruker Corporation, Karlsruhe, Germany), Before the observation the samples were polished at −130 °C using cryo-ultramicrotome (Leica EM UC7; Wetzlar, Germany). [9,10]. The rubber agglomerates size (distribution) in the samples was determined with the Image-Pro Plus 4.5 software [8]. The number-averaged diameter (*d*_n_) of the rubber agglomerates in each sample was calculated according to the diameters of at least 50 rubber domains in the corresponding AFM image, and the diameter of the rubber domains with irregular shape is determined as the average of their minimum and maximum dimensions. The volume-averaged diameter (*d*_v_), and the polydispersity index (*PDI*) were calculated with the *d*_n_ by using the equations previously used in L’Abee’s studies [8,13]. The thicknesses of PP ligaments (*ID*_poly_) were calculated with the *d*_n_ by using the equation proposed by Wu [8,14].

In addition, an S-4800 scanning electron microscope (SEM) supplied by Hitachi Co. (Tokyo, Japan) was used to further observe the tensile fractured surfaces of different samples. Before SEM observation, the samples were firstly brittle fractured by using liquid nitrogen, and then the fractured surface was gold-coated and observed.

#### 2.4.3. Crystallinity and Crystal Structures by Wide-Angle X-ray Diffraction (WAXD)

WAXD measurements were performed at room temperature on an X-ray diffractometer (D8 Advance, Bruker, Germany) with Cu-Kα radiation (λ = 1.5406 Å) generated from a copper source operating at a power level of 40 kV and 40 mA. The samples were scanned over the 2θ range of 5°–50° at a scan rate of 3°/min. The crystallinity (*X*_c_) and the crystal structure of the PP matrix in TPV were determined from the WAXD patterns according to the following equations [8]:

(4) Xc=(AcAtotal)ϕPP

(5) $$A_{c}=A_{\text{total}}-A_{\alpha }$$

where $\phi_{\text{PP}}$ is the volume fraction of the PP phase, *A*_c_ is the area under the crystalline peaks, $A_{\alpha }$ is the area of the amorphous halo, and *A*_total_ is the total area under the X-ray diffraction curve. The PP phase in the TPV can crystallize into different types of crystals, the α and β forms being the most common. The crystal fractions can be quantified by:

(6) $$X_{i}=\frac{A_{i}}{A_{\alpha }+A_{\beta }}$$

where *i* refers to a specific crystal structure (either α or β) and *A_i_* is the area of the corresponding crystalline peak. *A*_α_ and *A*_β_ were determined by using the Jade 6 software [8].

#### 2.4.4. Tensile Properties

The tensile properties of the EPDM/PP TPVs were measured by using a tensile apparatus (CMT4104, Shenzhen SANS Testing Machine Co., Ltd., Shenzhen, China) at a crosshead speed of 200 mm/min. The compression-molded films were punched to dumbbell-shaped tensile bars (25 × 6 × 1 mm^3^). Each sample was tested at least 5 times and the average value was taken. The tensile modulus, tensile strength, and elongation at break were determined from the stress-strain plots. The tensile modulus was obtained by calculating the slope of the stress-strain curve at the strain of 5%. In order to understand the mechanism for the microstructure-tensile properties of TPV, the deformation behaviors of the TPV samples were studied through observing the microstructure of the stretched samples with different elongation strains by using PF-AFM. The TPV products are usually used at a low strain (≤50%), where they can perform as traditional rubber with high elasticity. However, the stretched TPV samples after a large deformation are with a high permanent deformation and a small retraction because of the plastic deformation of the PP matrix. The stretched TPV samples we observed in this study are all elongated to a high strain (200%–800%), thus the high permanent deformations of the stretched TPV samples were almost the same as the elongation strains of the samples upon loading.

The cyclic stretching stress-strain curves of the samples were obtained by using the CMT 4104 tensile testing machine at a crosshead speed of 500 mm/min, and the largest strain was set at 50%. The hysteresis loss of samples at a strain of 50% was calculated by the ratio of the area of the loading-unloading loop to the area under the loading curve [15]. The remaining tensile strain of the sample after the tensile stress was recovered to 0 MPa was considered as the permanent deformation.

#### 2.4.5. Rheological Behavior

A strain-controlled rotational rheometer (AR-G2, TA Instruments, New Castle, DE, USA) was used in the parallel-plate geometry (diameter = 20 mm; gap = 1 mm). Frequency sweeps from 10^−1^ to 10^2^ rad/s were performed at 210 °C and a strain of 1.0%.

## 3. Results and discussion

### 3.1. Evolution of Degree of Crosslinking of TPVs during DV

**Figure 1:**
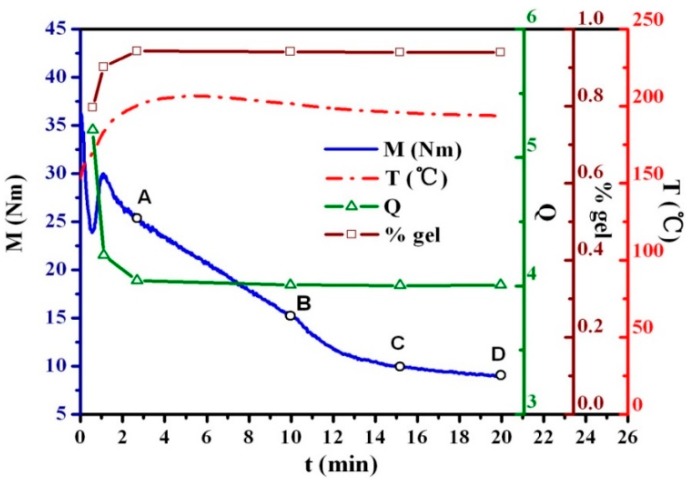
Variations of torque (*M*), temperature (*T*), swell ratio (*Q*) and rubber gel content (% gel) of the ethylene-propylene-diene monomer (EPDM)/polypropylene (PP) (60/40) blend during dynamic vulcanization (DV). Four samples (A, B, C, D) are selected according to the curve.

In order to study the influence of microstructure evolution of TPVs on their properties and avoid the influence of the degree of crosslinking of the rubber phase, the evolution in degree of crosslinking of the rubber phase in the EPDM/PP blends during DV was studied. The test samples were selected according to the torque (*M*)-time curves and temperature (*T*)-time curves (see Figure 1), representing the crosslinking of the rubber phase and the morphological evolution of the TPV [10]. The degree of crosslinking of the rubber phase in these samples were characterized by the swell ratio (*Q*) and the rubber gel content (% gel), and the results are shown in Figure 1. *Q* first decreases rapidly and then starts to level off with further increases in mixing time, whereas % gel first increases, reaches a maximum at a mixing time of 3 min, and then levels off. Thus, four samples (A, B, C, D) with the same degree of crosslinking were selected at the mixing times of 3, 10, 15 and 20 min. According to our previous studies, the phase inversion in the EPDM/PP blends occurred at the mixing time of 3 min (point A), indicating the formation of TPV [10].

### 3.2. Evolution of Size of Rubber Agglomerates during DV

**Figure 2:**
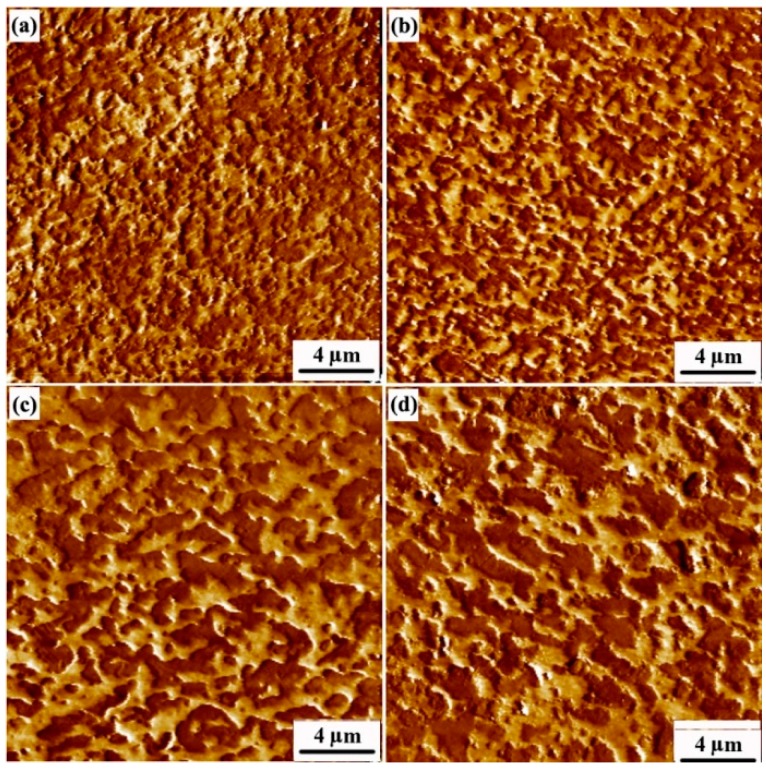
AFM phase images of EPDM/PP (60/40) TPV samples taken at different DV times: (a)–(d) represent the AFM images of samples A–D, respectively. (The lighter and darker regions represent the PP and EPDM phases, respectively).

Because the degree of crosslinking of the rubber phase is the same for samples A to D, the morphology of the rubber phase has the largest effect on their properties. PF-AFM was used to observe the microstructure of the compression-molded TPV samples, which were used for tensile properties and elasticity testing (Figure 2). The lighter and darker regions in Figure 2 represent the PP and EPDM phases, respectively. In sample A, the EPDM phase has already transformed into the dispersed phase in the continuous PP matrix, indicating that the phase inversion of the EPDM/PP blend has occurred [10]. These dispersed rubber particles formed during DV are actually the agglomerates of rubber nanoparticles, and the size of these agglomerates increases with increasing DV time due to the spontaneous agglomeration of the rubber nanoparticles [9,10]. We quantitatively characterized the size distributions of the RNAs and the thicknesses of the PP ligaments (*ID*_poly_), and the results are shown in Table 1. The number-averaged size of the RNAs (*d*_n_) increases significantly from A to D, and the corresponding volume-averaged diameter (*d*_v_) increases from 0.70 to 2.09 µm. The *PDI* decreases from 1.31 to 1.06, indicating that the average diameter of the RNAs increases, but the distribution of the diameters of the RNAs decreases with increasing DV time. Furthermore, *ID*_poly_ of sample A is 0.12 µm and it sharply increases to 0.41 µm till point D. These results show that the agglomerates of rubber nanoparticles and the rubber network in A are metastable, and they thus further agglomerate in the later stage of DV (C and D), leading to the deterioration of the rubber network, consistent with our previous results [10].

### 3.3. Evolution of Crystal Structure of PP

The crystallinity (*X*_c_) and the crystal structure of the PP phase in the TPVs also play a role in the properties of TPVs. Thus, we studied *X_c_* and the crystal structure of the PP phase of the compression-molded samples A to D by WAXD, and the results are shown in Table 2. We can see that samples A to D all have a *X*_c_ of about 43%, indicating that the DV time does not affect the *X*_c_ of PP in the TPV. Moreover, samples A to D contain both α PP and β PP crystals, and the fraction of β PP crystals decreases with increasing DV time. The formation of β crystals could be attributed to the nucleating effect of the crosslinked rubber particles [16,17]. The rubber particles maintained the oriented PP chains formed by concentration fluctuation at the interface during phase separation or shear stress during melt mixing. An increase in *d*_n_ results in a decrease in the surface area of the RNAs, thus the nucleation effect on the oriented PP chains deteriorates, and the fraction of β PP crystals decreases consequently.

### 3.4. Effect of Rubber Nanoparticle Agglomerationon Elasticity of TPV

**Figure 3:**
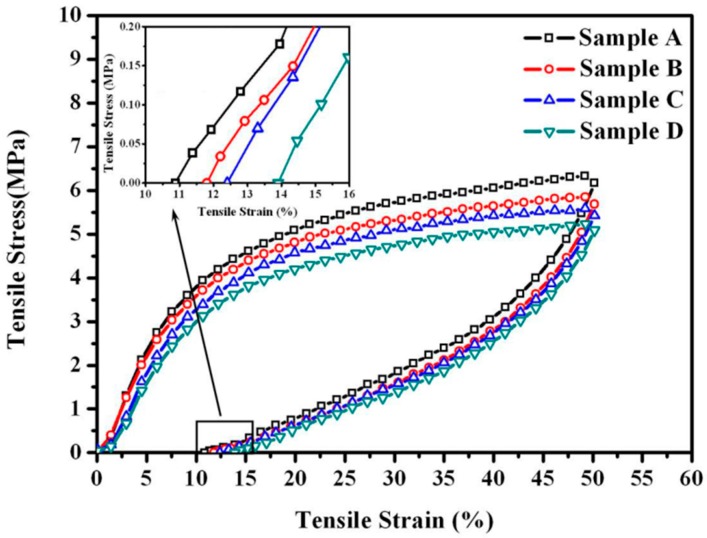
Tension-recovery stress-strain curves of EPDM/PP (60/40) TPV samples taken at different DV times.

**Figure 4:**
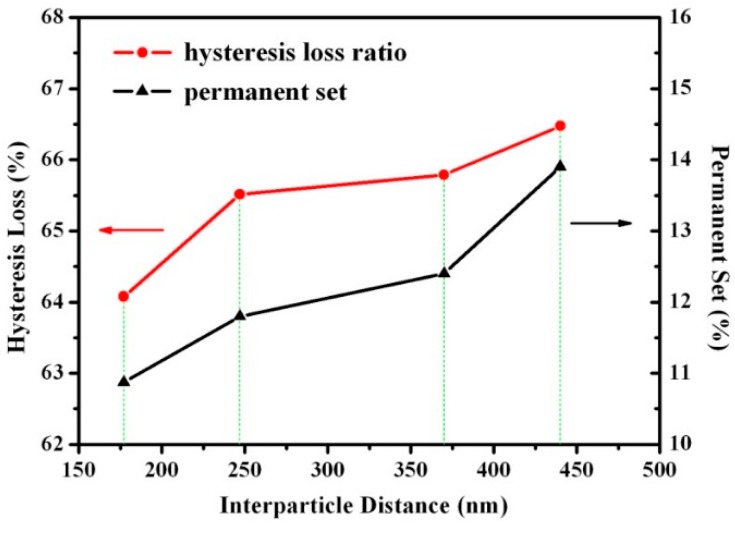
Hysteresis loss and permanent deformation as a function of interparticle distance (*ID_poly_*).

Hysteresis loss is widely used as an indication of the elasticity of elastomers [18]. A large permanent deformation caused by the slippage and internal friction between macromolecular chains can result in a high hysteresis loss [19]. It was reported that only at low strains (≤50%) did the matrix and the rubber phase in a TPV deform elastically and was the applied deformation recoverable [20]. Thus, samples A to D were stretched to the strain of 50% in the cyclic stretching tests. The stress-strain curves are shown in Figure 3, and the corresponding permanent deformations and hysteresis losses are summarized in Figure 4. We can see that the hysteresis losses and the permanent deformations of the samples are higher than those of traditionally vulcanized rubbers, indicating that TPVs have lower elasticity [21]. As *ID*_poly_ increases, the hysteresis loss and the permanent deformation of the TPV increases because the PP matrix of the TPVs is a semi-crystalline polymer, which deforms plastically and irreversibly [22]. In addition, these results indicate that the elasticity of TPV with RNAs with the diameter of 0.5–2 µm deteriorates with increasing *ID*_poly_, consistent with the variation of the elasticity of TPVs with rubber microparticles [8]. In other words, the elasticity of the TPV deteriorates with the increase in DV time. Thus, we can obtain a high elasticity of the TPV at a short DV time (Point A).

As mentioned above, the crystallinity of the PP matrix, the rubber content, and the degree of crosslinking of the rubber phase are all the same for samples A to D. Thus, the deterioration in the elasticity of the TPV with increasing DV time can be ascribed to the increase in the thicknesses of the PP ligaments (*ID*_poly_) and the deterioration of rubber network caused by the agglomeration of rubber nanoparticles during DV, as also demonstrated by the RPA (Rubber Process Analysis) results and disintegration results in our previous study [10]. The increase in *ID*_poly_ leads to a less effective localization of plastic deformation in the PP ligaments in the equatorial regions of the rubber particles, which can be explained by the simulation results of Boyce *et al*. [22,23]. In addition, the elasticity of the PP matrix was found to be inversely proportional to the cube root of the modulus of the PP plastic layer (Ep)13 when the modulus and the content of the rubber phase in the TPV both keep unchanged [8,24,25]. Since *X*_α_ remains almost constant while *X*_β_ decreases with increasing DV time, *E*p is expected to increase. Thus, the decrease in elasticity of the TPV with increasing DV time is partly ascribed to the crystal structure of the PP matrix.

### 3.5. Effect of Rubber Nanoparticle Agglomeration on Melt Processability

**Figure 5:**
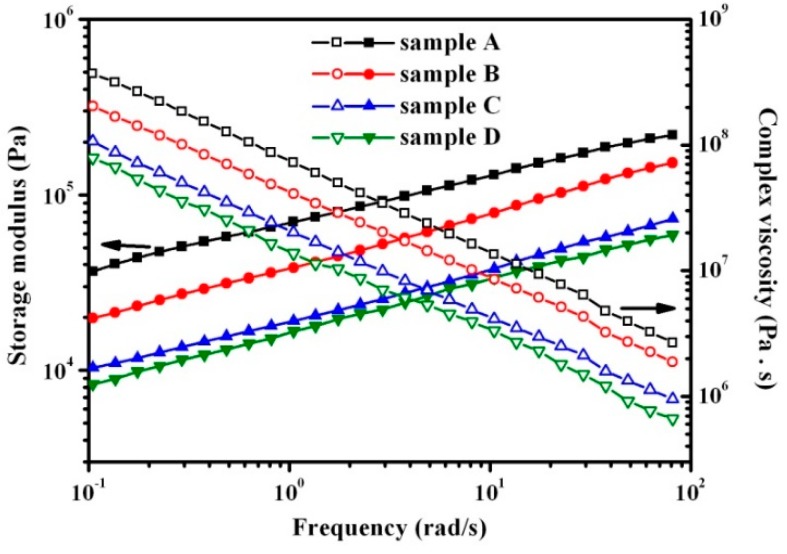
Storage modulus (*G’*) and complex viscosity (η*) at 210 °C as a function of angular frequency for samples A to D.

Figure 5 shows the complex viscosity (η*) and storage modulus (*G’*) as a function of the applied angular frequency (ω) at 210 °C for samples A to D. The slope of log (η*) *versus* log (ω) for these samples approaches −1 at low frequencies, indicating that these samples behave almost like an ideal elastic network under these conditions. Moreover, the high *G’* of these samples is similar to those of block copolymers and concentrated particulate composites, indicating that the dispersed RNAs form rubber network in TPV [26]. As the DV proceeds, η* and *G’* obviously decrease from samples A to D, indicating that the processability of TPVs is improved with the increase in DV time. This can be attributed to the increase in the thicknesses of the PP ligaments and the deterioration of the rubber network caused by the agglomeration of rubber nanoparticles in TPV, consistent with the effect of *ID*_poly_ on the processability of TPV with rubber microparticles 1–70 µm in diameter [8,27].

### 3.6. Effect of Rubber Nanoparticle Agglomeration on Tensile Properties

**Figure 6:**
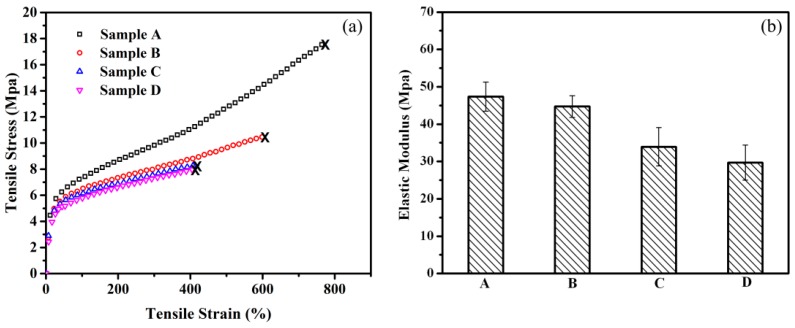
Tensile properties of EPDM/PP (60/40) TPV samples selected at different DV times: (**a**) stress-strain curves; (**b**) elastic modulus.

Figure 6 shows the tensile properties of samples A to D. All these samples deform homogeneously, and necking of the tensile bars is not observed. Figure 6a shows the stress-strain curves. The tensile strength (*TS*) of sample A is 18 MPa, and its elongation at break (ε) is as high as 780%. Both the *TS* and ε decrease with the increase in DV time, and the *TS* and ε of sample D are about half of those of sample A. Since the content of rubber phase and the crystallinity of the PP matrix both keep constant from point A to point D, the deterioration of the tensile properties of the TPV with increasing DV time should be mainly attributed to the variation of phase morphology, especially *d*_n_ and *ID*_poly_, which will be discussed in detail below. From Figure 6b and Table 1, we can see that the elastic modulus (*E*) decreases with increasing size of the RNAs (from samples A to D). This result is different from that reported by L’ Abee and Duin, in which the *E* of the TPV increases with the increase in *d*_n_ in the range of 1–70 µm [8]. The reason for this difference is that the diameter of the RNAs in EPDM/PP TPV we studied is in the 0.5–2 µm range, thus the interconnection between the dispersed RNAs and the rubber network are the most crucial factors influencing the *E* of the TPV. Despite the increase in the fraction of β crystals, which has a lower modulus than α crystals, in the PP matrix from samples A to D, the *E* of the TPV decreases because of the agglomeration of rubber nanoparticles and the deterioration of the rubber network. In short, we can simultaneously obtain a high *TS*, high ε, high *E*, and high elasticity of the TPV at an optimum DV time (sample A) by the achievement of a small *d*_n_, a thin *ID*_poly_ and a dense rubber network, but with a deterioration of processability.

**Figure 7:**
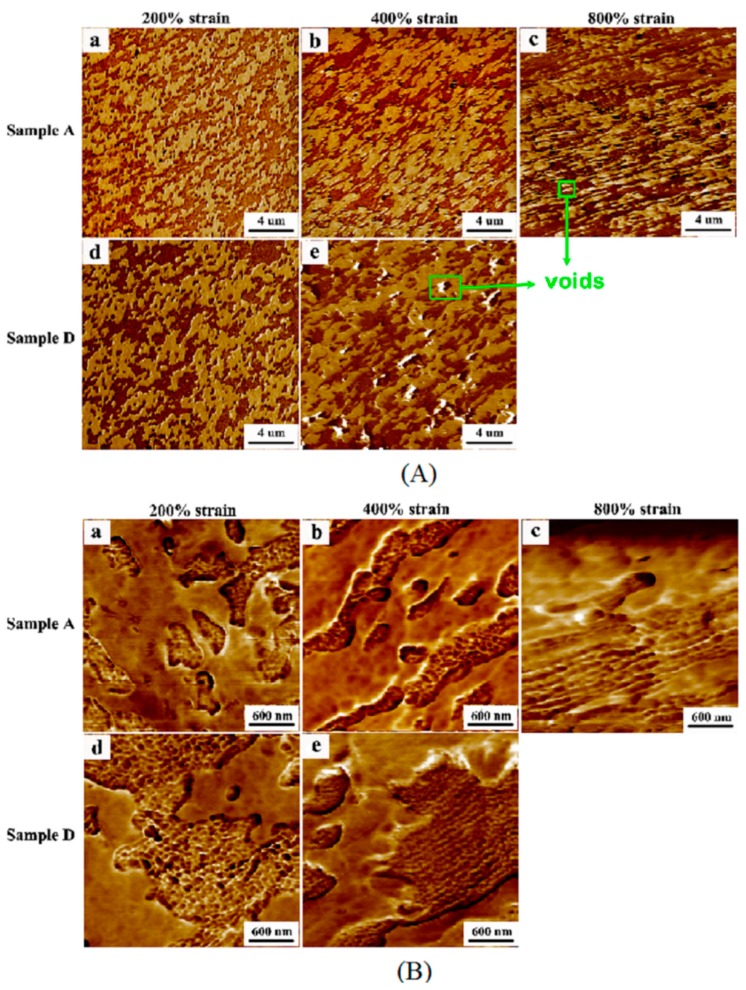
AFM images of elongated samples at different tensile strains (The darker and lighter regions represent the EPDM and PP phases, respectively.): (**A**) Low magnification; (**B**) high magnification. a, b, c are the AFM images of Samples A with strain of 200%, 400%, 800%, respectively; d and e are the AFM images of Samples D with strain of 200% and 400%, respectively.

To understand the deformation mechanism of the TPVs at different DV times, the PF-AFM images of the tensile bars of samples A and D at different tensile strains were obtained, and the results are shown in Figure 7. From Figure 7A, both the EPDM and PP phases are elongated during the deformation, and voids can be observed in the PP matrix in the broken tensile bars of sample A and D. Thus, the deformation behavior of TPVs at high strains is dominated by the formation of interlamellar voids in the semi-crystalline matrix, as reported in previous studies [2,8,18]. On the other hand, the voids in the broken sample D are much larger than those in the broken sample A, as shown in Figure 7A, because the number of RNAs is much larger in sample A than in sample D. Thus, the RNAs in sample A have a better suppression effect on the formation of interlamellar voids in the PP matrix [22]. Consequently, the increase in *d*_n_ and *ID*_poly_ during the DV reduces the *TS* and ε of the TPV (see Figure 2 and Table 1). Interestingly, the RNAs are oriented along the tensile direction in both samples A and D during stretching, and the degree of orientation increases with increasing tensile strain, as shown in Figure 7B. The orientation of the RNAs is caused by the slippage of the rubber nanoparticles during stretching, which can absorb energy, and further slows down the development of voids or cracks, and thus facilitates the increase in the *TS* and ***ε*** of the TPV [28]. The smaller and more numerous RNAs in TPV sample, the more energy can be absorbed during the orientation because of the larger interfacial area between the RNAs and PP, leading to a higher *TS* and ε. In addition, the decrease in *X*_β_ during DV can also contribute to the decrease in the *ε* of the TPV, but the effect of the crystal structure on the *TS* and ε of the samples is negligible because the change in *X*_β_ is small during DV (see Table 2) [29].

**Figure 8:**
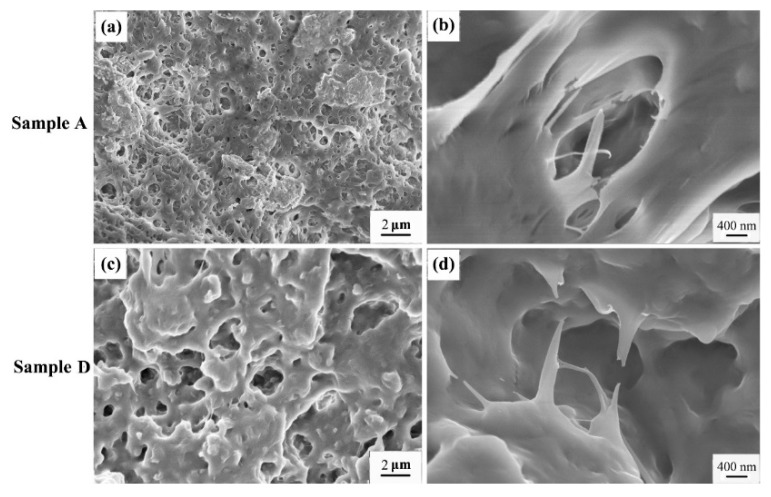
SEM micrographs of tensile fractured surfaces of the samples A and D.

In addition, the morphologies of the tensile-fractured surfaces of samples A and D were observed by using SEM, and the results are shown in Figure 8. We can see many cavities formed when the RNAs are pulled out from the PP matrix, thus the size of cavities can reflect the size of the RNAs. As expected, the cavities on the fractured surface of sample A are much smaller and much more numerous than those of sample D, consistent with the AFM results (see Figure 2). Furthermore, both samples undergo ductile fracture. However, stress whitening is observed in sample A, but not in sample D, again indicating that the toughness of sample A is higher than that of sample D, which is consistent with the result demonstrated by the stress-strain curves of sample A and D.

## 4. Conclusions

The microstructure-properties relationships of EPDM/PP TPV during DV were revealed, especially the effect of the *d*_n_, *ID*_poly_, and rubber network on the mechanical properties, elasticity, and processability. The conclusions obtained with rubber nanoparticle agglomerates (RNAs) 0.5–2 μm in diameter support L’Abee’s conjecture about the properties-processing balance for TPVs with sub-micron rubber particles size [8]. We simultaneously obtained EPDM/PP TPV with high *TS*, ε, *E*, and elasticity by the achievement of a smaller *d*_n_, a thinner *ID*_poly_ and a denser rubber network by controlling the DV time, but with a deterioration of processability. The large change in the rubber network caused by the agglomeration of rubber nanoparticles results in a different effect of the *d*_n_ and *ID*_poly_ on the *E* of the TPVs from that of the TPVs with rubber microparticles 1–70 μm in diameter reported previously. A mechanism based on the deformation behavior of TPVs with RNAs during stretching for the achievement of good mechanical properties was revealed. Interestingly, we found that the RNAs are oriented along the tensile direction during stretching. The TPV samples with smaller and more numerous RNAs can absorb more energy and can slow down the development of voids or cracks more effectively, thus increasing in the tensile strength and elongation at break of the TPVs.

## Figures and Tables

**Table 1:** Rubber particle sizes and size distributions of TPV samples.

Number	*d*_n_ (µm)	*d*_v_ (µm)	*PDI*	*ID*_poly_ (µm)
Sample A	0.53 ± 0.11	0.70	1.31	0.12
Sample B	0.72 ± 0.25	0.91	1.26	0.16
Sample C	1.46 ± 0.24	1.58	1.08	0.31
Sample D	1.96 ± 0.22	2.09	1.06	0.41

**Table 2:** Crystallinities and crystal structures of selected TPV samples.

Number	*X*_c_ (%)	*X*_α_ (%)	*X*_β_ (%)
Sample A	42.4 ± 1.3	37.7	4.7
Sample B	43.6 ± 0.9	41.5	2.1
Sample C	42.7 ± 1.2	41.8	0.9
Sample D	42.3 ± 2.1	41.5	0.8
